# What characteristics of provider payment mechanisms influence health care providers’ behaviour? A literature review

**DOI:** 10.1002/hpm.2565

**Published:** 2018-07-08

**Authors:** Jacob S. Kazungu, Edwine W. Barasa, Melvin Obadha, Jane Chuma

**Affiliations:** 1 Health Economics Research Unit, KEMRI Wellcome Trust Research Programme, Nairobi, Kenya; 2 Nuffield Department of Medicine, Oxford University, Oxford, UK; 3 Kenya Country Office, World Bank Group, Nairobi, Kenya

**Keywords:** attributes, characteristics, health care provider response, provider payment mechanisms, provider payment methods

## Abstract

**Background:**

Provider payment mechanisms (PPMs) create incentives or signals that influence the behaviour of health care providers. Understanding the characteristics of PPMs that influence health care providers’ behaviour is essential for aligning PPM reforms for improving access, quality, and efficiency of health care services. We reviewed empirical literature that examined the characteristics of PPMs that influence the behaviour of health care providers.

**Methods:**

We systematically searched for empirical literature in PubMed, Web of Science, and Google Scholar databases and complemented these with physical searching of the references of selected papers for further relevant studies. A total of 16 studies that met our inclusion and exclusion criteria were identified. We analysed data using thematic review.

**Results:**

We identified seven major characteristics of PPMs that influence health care providers’ behaviour. Of these characteristics, payment rate, the sufficiency of payment rate to cover the cost of services, timeliness of payment, payment schedule, performance requirements, and accountability mechanisms were the most important.

**Conclusions:**

Our review found that health care providers’ behaviour is influenced by the characteristics of PPMs.Provider payment mechanism reforms that optimally structure these characteristics can elicit required incentives for access, equity, quality, and efficiency in service delivery among health care providers towards achieving universal health coverage.

## 1 Introduction

Universal health coverage (UHC) is a key health agenda in the era of sustainable development goals.^1^ Universal health coverage requires that all individuals in a population have access to needed health services of good quality and are protected from financial ruin. Moving towards UHC however requires health system reforms that are aligned to UHC goals.^2–4^ While UHC reforms have traditionally focused on how to mobilise additional resources and establishing resource pools especially in low and middle-income countries, there is a growing recognition of the need to prioritise health care purchasing reforms.^4,5^ Health care purchasing entails decisions in three main action areas: what health services to buy (benefit package), whom to buy from (choice of health care providers), and how to buy them (provider payment mechanisms, price, and other contractual arrangements).^6–8^ Provider payment mechanisms (PPMs) are critical to attaining UHC goals because they generate incentives and signals for improving access, quality, and efficiency of health care services among health care providers.^9,10^

Provider payment mechanisms refer to the way in which funds are transferred from a purchaser (the organisation transferring funds such as a Ministry of Health or a health insurance firm) to a health care provider.^7,11^ Table 1 provides a summary of common PPMs. The suitability of a PPM is highly context-specific and dependent on the availability of governance and institutional arrangements to regulate and enforce them. Most countries use a combination of PPMs to reimburse different services or service packages,^9,14–17^ as each method has advantages and disadvantages. For example, family doctors in the UK are mostly paid on a capitation basis but also receive performance-based payments.^18,19^

Empirical evidence supports the theoretical assertion that different PPMs create incentives or economic signals that influence provider behaviour.^9,12^ For instance, findings from 2 recent reviews that evaluated the impact of different PPMs on health outcomes and the overall quality of care provided by health care providers showed that the quantity of health care services (hospitalisations, the number of diagnostic and curative services, and clinical consultations—number and time) reduced under capitation but increased under fee for service.^9,20^ Similar findings were shown in a real effort experiment where fee for service had the highest quantity of output, salary recorded the least, while high quality was achieved when health care providers were paid by capitation or salary but least when fee for service was used.^21^ Moreover, Krasnik et al^22^ showed that higher rates of specialist and hospital referrals were observed when providers were paid on a capitation basis.

Although several studies have examined how health care providers respond to different PPMs and their impacts on health care outcomes, only a few studies explore the specific characteristics of PPMs that health care providers respond to. Understanding the characteristics of PPMs that influence health care providers’ behaviour is essential to inform decisions for targeted PPM design and reforms. We carried out a thematic review of empirical literature to fill this important gap in evidence and inform the design of PPMs, as countries reform their health systems for UHC.

## 2 Methods

### 2.1 Literature search

We searched literature in February 2018 in PubMed, Web of Science, and Google Scholar. We utilised the following search terms to locate relevant literature: “Characteristics” OR “Features” AND “Provider payment mechanisms” OR “Provider payment Methods” OR “Payment methods” OR “Payment mechanisms” OR “Remuneration mechanisms” OR “Compensation method” OR “Budgets” OR “global budgets” OR “line item budgets” OR “Capitation fee” OR “capitation” OR “fee for service” OR “fee-for-service” OR “FFS” OR “case-based reimbursement” OR “pay for performance” OR “p4p” OR “Physician Incentive Plans” OR “Mixed payment systems”. We also used a snowballing technique of searching for relevant literature from the reference list of included studies. Our search comprised of all published studies up to the time of the literature search (February 28, 2018).

### 2.2 Eligibility criteria

Only full-text papers that reported empirical research on the experiences and/or perceptions of health care providers with regards to the characteristics of PPMs were included. We included only studies published in English language and those whose respondents were direct health care providers (such as doctors or nurses) while excluding studies describing the characteristics of PPMs from a patients’ and/or policymakers’ perspective. We screened the identified studies in three stages: (1) screening by title, (2) screening by abstract, and (3) screening by reading the full text. We finally excluded studies that examined the incentives that health care providers may have under different PPMs. Two authors independently reviewed all abstracts and full-text formats of the studies. After screening, data were extracted from the remaining studies.

**Figure 1:**
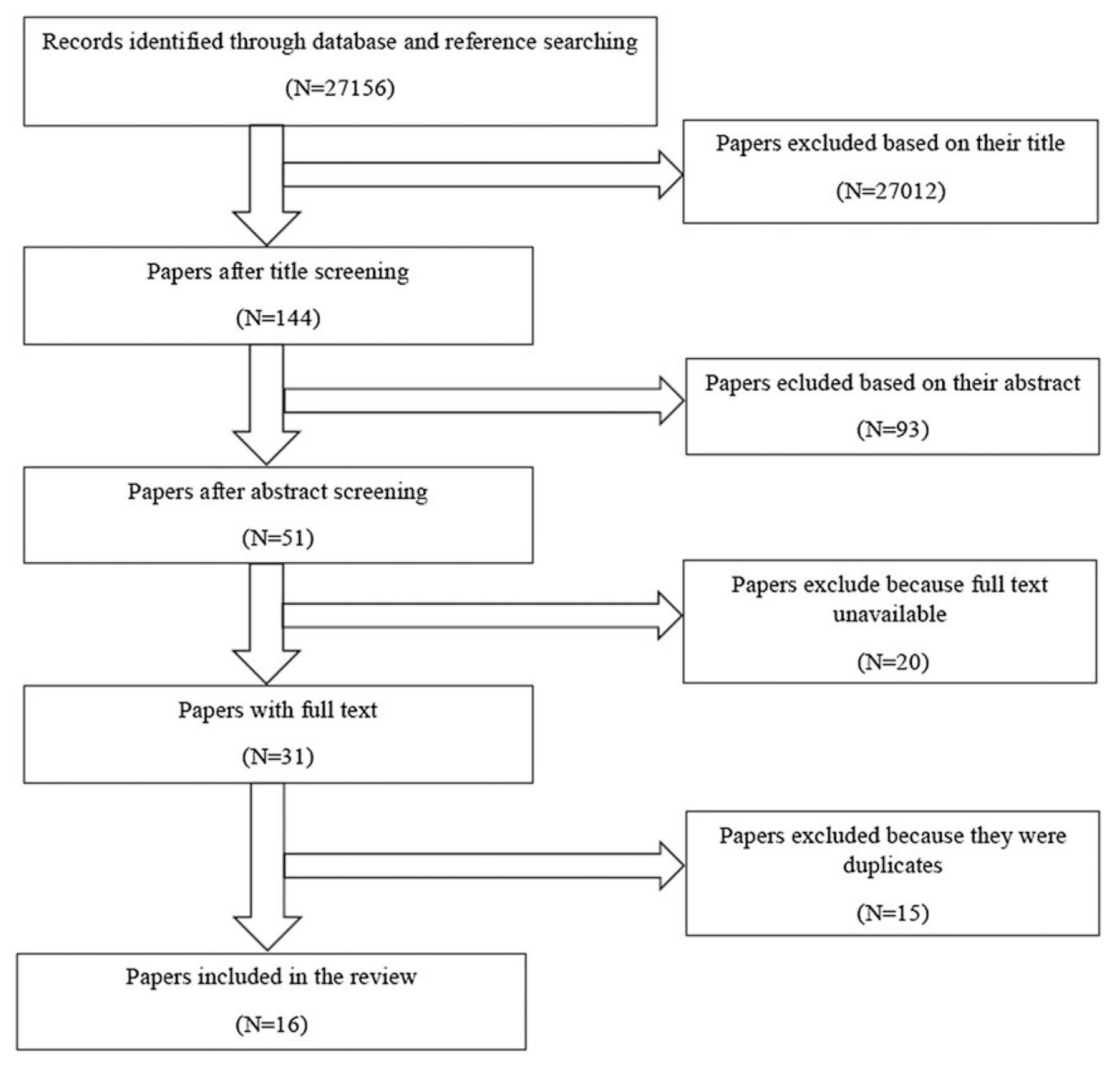
Screening process to obtain selected papers

We identified 27 156 references after the first search. Of the 27 156 studies, 27 105 articles were excluded after a review of titles and abstracts because they were either not empirical or did not examine the characteristics, experiences, and/or perceptions of health care providers with regards to PPMs. Twenty more articles were excluded because of lack of full text (Figure 1). A further screening eliminated 15 more articles for being duplicates. The review finally comprised of 16 articles (Table 1).

### 2.3 Characteristics of selected papers

Table 2 shows the number and characteristics of the selected papers. Despite the omission of a time restriction to our search, only 16 studies met our criteria for inclusion. This highlights the fact that empirical studies focusing on the characteristics of PPMs from a health care providers’ perspective remains fairly low. Out of the 16 studies, 6 studies were conducted in the USA. Ghana and Taiwan contributed 2 studies each, while Nigeria, Tanzania, Rwanda, Netherlands, and Burkina Faso contributed a study each (Table 2). Additionally, 1 paper reported results from a multicountry study conducted in Ghana and Kenya. Capitation, fee for service (FFS), and payment for performance (P4P) were the most reported types of PPMs (Table 2). These payment mechanisms were paid to either individual providers such as doctors (health workers) and/or organisation providers such as hospitals. Bonuses and case-based (episode-based and case-mix) payment mechanisms were only reported in studies conducted in the USA. Global budget payment and Diagnostic Related Group payment were discussed in studies from Taiwan and Ghana respectively.

### 2.4 Quality assessment

Table 3 outlines the findings from the quality assessment. We applied the Critical Appraisal Skills Programme tool.^39^ Critical Appraisal Skills Programme uses a standardised checklist which contains screening questions to evaluate the appropriateness, trustworthiness, and objectivity of the findings described in the research articles under review.^39,40^

All studies included in this review had clear statements about the objectives, methodology, research design, data collection procedures, and analytical approaches and either contributed to existing knowledge, proposed new research areas, or discussed the transferability of their findings to other contexts. However, most studies scored poorly in two areas: (1) ability to adequately identify areas of researcher subjectivity during the study design and (2) providing evidence on ethical approval and considerations to informed consent and confidentiality. We observed that (1) there existed differences in writing the methods section where different writing practices and styles were adopted by different researchers and (2) most of the studies conducted analyses of secondary data where such studies might not have been subjected to ethical approval. Despite a poor score in these 2 areas, we opted to include all the papers in our review as they remained relevant to our review objective.

### 2.5 Synthesis of selected papers

We carried out a thematic assessment of the identified papers by ensuing 4 key steps: (1) a first reading through the identified articles to familiarise with the studies while identifying key thoughts/concepts about PPMs, (2) coming up with a coding framework, (3) a thorough second reading of the identified articles and matching identified contents from each article onto the coding framework, and (4) recording the matched data and analyzing by generating key characteristics from these emergent concepts in an explanatory stage where results from the selected papers were incorporated into clear themes. The coding process was conducted manually in MS Excel.

## 3 Results

### 3.1 Characteristics of provider payment mechanisms

Table 4 shows the major themes (hereafter called characteristics) emanating from this review. We identified 7 major characteristics: payment rate, accountability mechanisms, payment schedule, performance requirements, bundling of services, the sufficiency of payment rates to cover the cost of services, and timeliness of payment.

Out of the 16 papers included in our review, payment rate was identified in over 50% of the studies (9/16) (Table 3). In 5 studies, health care providers identified accountability mechanisms and payments based on performance and identified some performance indicators they considered important. Payment schedule, bundling of services (where services are bundled and paid for together as a package), and timeliness in payment was each reported in 3 studies.

### 3.2 Sufficiency of the payment rate

Payment rate is at the core of any PPM. Ensuring that payments can adequately cover the cost of services offered is a crucial characteristic of any PPM. Payment rates act as the starting point for negotiations between health care providers and purchasers.^41^ The reviewed literature suggests that health care providers preferred a PPM that had a higher payment rate compared to one whose rate was low on average.^23,27–31,33,37,38^ Sufficiency of payments to cover the cost of services was identified by health care providers in 4 studies. For example, Agyepong et al^36^ highlighted that providers in Ghana considered payment rates as inadequate to cover the costs of inputs needed to manage a patient. Also in Ghana, health care providers expressed concerns that the per capita payment rate was too low and demanded an increase.^37^ Even after a 22% increase in the payment rate, some providers still felt the rate was inadequate. This corroborates with Ellis et al^42^ who suggested that for almost any payment system, payment rates are a key factor to providers and often reduce incentives for quality if they are set too low. A higher payment rate would increase health care providers’ revenues^43^ thus relaxing budget constraints and therefore enabling them to invest more in service provision such as to increase the number of staff. For example, Feng et al^30^ observed that higher Medicaid payment rates increased total staffing levels in nursing homes, while Harrington et al^31^ also found that higher Medicaid reimbursement rates were associated with high registered nurses staffing levels in the USA.

### 3.3 Accountability mechanism

Accountability mechanisms refer to answerability or reporting requirements associated with PPMs. These include documentation required for filing claims, audits, performance monitoring, and other reporting requirements. Olafsdottir et al,^32^ exploring the potential of P4P in addressing barriers to attaining performance targets in Tanzania, showed that health care providers regarded supervision and monitoring (especially monitoring with feedback) as an important factor for a good P4P mechanism that would foster its smooth implementation. For a performance-based payment mechanism, monitoring with feedback was mainly attributed to providing health care providers with required information for assessing their status while identifying areas to improve to achieve performance targets.^44^

### 3.4 Payment schedule

This characteristic refers to the period/frequency of payment. Health care providers preferred shorter intervals between payments.^33^ For instance, Robyn et al^33^ found that health workers were more likely to select a capitation payment option where payments were made 4 times per year (quarterly) than annual payments. While being paid annually, health workers experienced depletion of funds which posed a challenge to the availability of important commodities such as drugs. A relatively shorter interval (such as biannually or quarterly) would boost a continuous flow of funds that will aid budgeting and purchasing of necessary commodities. Similarly, Chen et al^28^ found that physicians significantly preferred a P4P payment bonus made every 6 months compared to an annual payment. A key utility of shorter payment schedules was in avoiding financial deficits leading to stock-outs of essential commodities, which was considered as a prerequisite component for budgeting purposes.

### 3.5 Payment based on performance

Performance requirements was also identified as an important characteristic for reimbursement methods.^25–28,34^ Health care providers preferred payments that are based on performance. Performance-based payment was attributed to the motivation of health workers to enhance quality and access, contain costs while maintaining safety in health care service delivery.^45^ However, there were variations as to whether performance should be based on quantity, quality, and/or other process or outcome measures. In the Reschovsky study^25^ that examined (among other things) how compensation methods influence physician perceptions about whether monetary incentives are for increasing or decreasing services to patients, physicians most often cited productivity—an outcome measure—as the main factor influencing their reimbursements. Productivity was cited to pressure physicians to increase the quantity of services to patients to achieve higher performance outcome-based performance targets and higher revenues. On the other hand, Alqasim et al^26^ found that in as much as the payment rate based on performance was important, physicians expressed that the performance indicators need to focus on quality and organisational performance rather than individual performance. This was because quality measures are often difficult to assess at an individual level, and some activities (such as surgical activities) have overlapping roles.

### 3.6 Bundling of services

Bundling of services refers to aggregating of 2 or more services and paying for them as a group, as opposed to billing and reimbursing for each individual service separately. For example, services can be bundled together into a group such as outpatient services. Health care providers opposed PPMs where services were bundled. The opposition to the bundling of services was mainly attributed to the financial risk of incurring losses especially when patients require a wide range of services within the bundle. For example, Federman et al^29^ found that nearly 70% of physicians opposed bundling of payments in the USA because of the fear that the revenues generated may not reflect the costs incurred. While describing per-capita (capitation) payments reforms with regards to changes in primary care maternal services in Ghana, Koduah et al^37^ found that providers needed maternity services excluded from the bundle of services covered under the capitation payment method as they will be incentivised to reduce service inputs to contain costs. Agyepong et al^36^ found that bundled payment was a disincentive for health care providers to perform extensive diagnostic investigations in Ghana’s Diagnostic Related Group as extensive diagnostic tests are often expensive and the bundled payments are considered too little to adequately cover this cost.

### 3.7 Timeliness of payment

The importance of timely payments to health care providers was a frequent characteristic mentioned in 3 of the selected papers.^23,38,46^ The utility of timeliness of payments was for budgeting purposes and smooth provision of services. Timely payments made it easier for health care providers to plan and purchase commodities and pay employees and suppliers in time. For instance, Agyepong et al^36^ examined the effect of PPMs on health providers’ motivations and behaviour in Ghana and found that while payment rate was an important factor, timeliness in payment was the most important factor as it ensured financial predictability promoting a smooth running of hospitals and motivating health workers. In a multicountry study that explored the knowledge of private health care providers with the National Hospital Insurance Fund and National Health Insurance Scheme in Kenya and Ghana respectively, Sieverding et al observed that health care providers experienced delays in payments of 6 to 8 months.^38^ Consequently, delays in payments not only affected the availability of resources within the facilities especially medicines but also delays in settling employees’ salaries and suppliers’ bills. Elsewhere,^37^ health care providers had to suspend services to the National Health Insurance Scheme enrolees because of delayed payments.

## 4 Discussion

Our review highlights several characteristics that influence health care providers’ behaviour and are key to the design and reform of PPMs. First, the sufficiency of the payment rate to cover the cost of services was the most recurrent theme across the empirical literature on the characteristics of PPMs. As Perry et al highlighted, payment rates that adequately cover the cost of services increase health care providers’ revenues/income.^43^ With high and more stable revenues, health care providers can adequately plan with less budgeting constraints making not only resources (such as medicines, health workers) more available but also improve their performance. As providers are income-motivated,^47^ incorporating more generous payment rates in PPMs may motivate them to deliver high quality, efficient, and equitable health care services.

Second, it is not surprising that timeliness of payment influences health care providers’ behaviour. When payments are made in time, health care providers have an opportunity to budget for the funds and ensure necessary inputs such as medicines are available.^48^ However, delays in receiving payments affect the ability of health care providers to run facilities smoothly which makes them either underprovide services, refer patients to other facilities, stop providing services, or informally charge insured patients.^46,49^ These responses are not in line with the UHC goals of access, equity, quality, and efficiency of health care services with a focus on financial protection.

Third, payment schedule was another important factor influencing health care providers’ behaviour. While payment schedule is inter alia dependent on the type of a PPM, it has been shown to vary in the interval between monthly to annual payments.^50^ With respect to this range, however, our findings show that a shorter interval payment schedule is more preferred by health care providers compared to a longer one for several reasons: First, borrowing from the law of diminishing marginal returns, it can be argued that payment schedules with short intervals generate higher utility than less frequent payment which is characterised by longer interval payment schedules. Consequently, while acknowledging the low amounts and administrative burden of extremely frequent payments, Khullar et al^51^ suggested that higher utility is gained from, for instance, 12 monthly payments of $100 than a single $1200 payment made annually as individuals often reset their point of reference after every payment. Second, health care providers often cannot accurately predict the quantity of essential inputs (drugs, gloves, needles, etc.) needed over the period covered by the payment, as resources may be depleted during this period.^52^ Third, providers depend on these payments to, for instance, settle employees’ salaries and repair facilities. For example, FFS payments are often characterised by retrospective payments—where health care providers are paid for each individual service after services have been delivered.^11,53^ Consequently, longer waiting periods may hinder service delivery.

Fourth, while bundling of services and paying for them as a package has been shown to be an efficient way of reimbursing health care providers,^54^ often, health care providers oppose such PPMs. This may be because bundling exposes health care providers to greater financial risk^54^ and uncertainties on how to effectively control costs while providing all needed and high-quality services.

Fifth, health care providers respond to accountability requirements and to performance indicators because monitoring not only imposes checks on health care providers but also provide avenues for providers to get feedback on important aspects to improve on (such as claims process).^24,44^ This is particularly important when payments are based on performance. Health care providers view performance as a measure of their ability to provide services and a means to increase their revenues.^55,56^ Evidence suggests that financial rewards such as bonuses resulting from P4P is a key factor influencing provider behaviour.^51^ Furthermore, monitoring serves as an audit function to guard against gaming and overpayment.^44^ Importantly, performance-adjusted payment rates have been shown to motivate health care providers to align their health care services (outcomes) with the performance requirements, especially quality.^57^

### 4.1 Limitations

We acknowledge that there is a likelihood that we might have missed to include some studies in our review. We, however, minimised this by searching more than 1 database and searching the references of included studies. Furthermore, while getting all studies was important for this review, we aimed at interpreting findings rather than predicting as noted by Thomas et al.^58^

## 5 Conclusions

Understanding the characteristics of PPMs that influence health care providers’ behaviour is integral to designing or reforming PPMs that are aligned with the goals of universal health coverage. To our knowledge, this is the first review that examines the characteristics of PPMs that elicit responses from health care providers. Consequently, it is imperative to incorporate these characteristics in payment reforms with a view to not only elicit required incentives for improving access, quality, and efficiency of health care services but also with a view to striking a balance between health care provider satisfaction and the viability and sustainability of the payment mechanism. Our review presents the characteristics of PPMs considered important by health care providers, however, this does not show their relative importance. Examining the relative importance of each attribute would inform the trade-offs that health care providers are willing to make and therefore provide adequate information for tailoring payment reforms. Stated preference elicitation methods such as discrete choice experiments could be used to elicit preferences of health care providers for these attributes to achieve contextualised PPMs which generate the right incentives for improving access, quality, and efficiency in health care service delivery among health care providers.

## Figures and Tables

**Table 1:** A description of the main provider payment mechanisms

Provider Payment Mechanisms	Definition
Global budget	A prospective payment where health care providers are given an amount of money to spend, with total flexibility on how and what to spend on, to deliver an agreed-upon set of services
Line-item budget	A prospective payment where providers receive a given amount of money to spend on specific itemised services. The budget is not flexible, and expenditure must follow line items, unless with prior authorisation from relevant authorities
Fee for service (FFS)	A retrospective activity-based reimbursement method where health care providers are reimbursed for each individual service provided
Capitation (per capita)	A payment method where providers receive a fixed amount of money prior to service delivery, to provide agreed services for each registered individual over a fixed period
Per diem	Health care providers are paid a fixed amount for given services per day
Case-based (eg, diagnosis-related groups)	Providers are paid a fixed amount per case such as for each diagnosis, admission, or discharge
Pay for performance	Involves paying health care providers on the basis of the providers meeting certain performance thresholds based on predetermined measures

Sources: Adapted from Cashin et al,^12^ Langenbrunner et al,^11^ and Rosenthal et al.^13^

**Table 2:** Characteristics of selected papers

Author	Country	Study Objective	Provider Payment Mechanisms (PPMs) Discussed	Characteristics of PPMs Identified
Mohammed et al^23^	Nigeria	To use health care providers’ perspectives to evaluate the factors influencing optimal resource use domains	Capitation and fee for service	Payment rate, monitoring or accountability, and payment schedule
Hsu et al^24^	Taiwan	To examine whether a global budgeting compensation policy moderates the medical benefits claimed between 2000 and 2008	Global budget and fee for service	Accountability mechanisms
Reschovsky et al^25^	USA	To examine how payment methods affect physician beliefs of whether their overall financial motivations are to increase or decrease services to patients	Bonuses, capitation, and fee for service	Payment based on performance/productivity
Alqasim et al^26^	Netherlands	To assess the views, knowledge, and experience of Dutch physicians with regard to the general objectives and values of the pay-for-performance (P4P) system	P4P	Accountability and payment based on performance
Basinga et al^27^	Rwanda	To assess how performance-based payment of health care providers affect the use and quality of child and maternal care services in health care facilities in Rwanda	P4P	Payment rate, unit of payment, payment based on performance/productivity
Chen et al^28^	Taiwan	To determine the most important characteristics for designing a diabetes P4P programme in Taiwan	P4P	Payment rate, accountability mechanisms, payment schedule, and payment based on performance
Federman et al^29^	USA	To evaluate physicians’ opinions on the approaches for reforming physician payment methods while promoting quality of health care and containing costs	Bonuses and case-based payment	Sufficiency of payment rates to cover the cost of services and bundling of services
Feng et al^30^	USA	To examine the effect of different reimbursement methods on staffing levels in nursing homes in the USA	Case-mix reimbursements	Payment rate
Harrington et al^31^	USA	To examine the association between Medicaid payment rates and nursing staffing levels in nursing homes in the USA	Case-mix reimbursements	Payment rate
Olafsdottir et al^32^	Tanzania	To describe the contextual setting in which P4P was introduced in Tanzania and examine how P4P can address system limitations to meeting performance targets	P4P	Accountability mechanisms
Robyn et al^33^	Burkina Faso	To examine community-based health insurance scheme provider reimbursement characteristics that impact health care workers’ stated preferences for reimbursement mechanisms	Capitation	Payment rate, payment schedule, and sufficiency of payment rate to cover the cost of services
Tufano et al^34^	USA	To examine the perceptions of physician practising in medical groups and leaders on the association between physician reimbursement and physicians’ productivity	Capitation, production-based compensation, and salary	Payment based on performance/productivity
Wang et al^35^	USA	To examine pharmacists’ acceptable compensation for providing medication therapy management services	Not discussed	Payment rate
Agyepong et al^36^	Ghana	To describe the impact of provider payment mechanisms on provider motivations and behaviour related to the delivery of health care services to insured clients in Ghana	Ghana Diagnostic Related Group	Bundling of services, sufficiency of payment rate to cover the cost of services, and timeliness of payment
Koduah et al^37^	Ghana	To understand the process of health policy agenda setting, formulation, and implementation in Ghana	Capitation	Payment rate
Sieverding et al^38^	Ghana and Kenya	To explore private health care providers’ perceptions of and experiences with the National Health Insurance Scheme in Ghana and the National Hospital Insurance Fund in Kenya		Timeliness of payment, and payment rate

**Table 3:** Quality assessment checklist

Appraisal Criteria	Yes	Somewhat	No/Not Clear
1. Does article have a clear statement of the objectives?	16		
2. Does the methodology adequately help achieve the research objectives?	16		
3. Was the study design suitable to achieve the research objectives? Was there a justification for the study design?	16		
4. Were study participants recruited appropriately? Does the researcher provide a clear explanation of how the study participants were selected and why they were suitable?	14	1	1
5. Does the data collection approach appropriate to answer the research question? Was the data collection location justified? If it is clear how data were collected? Were data collection methods clear?	16		
6. Has the relationship between the researcher and the participants been adequately considered? Researcher reflexivity and potential partiality during the formulation of research questions or data collection?	7		9
7. Did the researchers consider ethical issues before conducting the study? Are issues on informed consent and confidentiality adequately addressed? Did the researchers seek ethical approval?	9	1	6
8. Was there adequate rigor during data analysis? An explicit explanation of how the analysis was conducted? A clear statement of how themes/categories were developed Are there proper considerations to inconsistent findings?	16		
9. Are findings reported clearly? Explicit findings An adequate discussion of evidence for and against the researcher arguments The credibility of finds (triangulation, respondent validation, more than 1 analyst), findings are discussed in relation to the original research question)	15	1	
10. How valuable is the research? The researcher explains how the study contributes new knowledge or adds to existing knowledge. Have researchers identified new areas for future research? Are there clear explanations about how the findings can be applied to other settings?	16		

**Table 4:** Main characteristics of provider payment mechanisms

Characteristic	Studies
Payment rate	Mohammed et al^23^ Basinga et al^27^ Chen et al^28^ Federman et al^29^ Feng et al^30^ Harrington et al^31^ Robyn et al^33^ Sieverding et al^38^ Koduah et al^37^
Sufficiency of payment rates	Agyepong et al^36^ Federman et al^29^ Wang et al^35^ Robyn et al^33^
Accountability mechanism	Hsu et al^24^ Mohammed et al^23^ Alqasim et al^26^ Chen et al^28^ Olafsdottir et al^32^
Payment schedule	Chen et al^28^ Hsu et al^24^ Robyn et al^33^
Performance indicators	Reschovsky et al^25^ Alqasim et al^26^ Basinga et al^27^ Tufano et al^34^ Chen et al^28^
Bundling of services	Federman et al^29^ Agyepong et al^36^ Koduah et al^37^
Timeliness of payment	Agyepong et al^36^ Mohammed et al^23^ Sieverding et al^38^

## References

[1] Association WH (2010). The World Health Report 2010-health systems financing: The path to universal coverage.

[2] Kutzin J, Cashin C, Jakab M (2010). Implementing health financing reform.

[3] Durairaj V, D’Almeida S, Kirigia J (2010). Health systems financing, the path to universal coverage. Ghana’s Approach to Social Health Protection. Backgr Pap.

[4] Kutzin J (2013). Health financing for universal coverage and health system performance: Concepts and implications for policy. Bull World Health Organ.

[5] Busse R, Figueras J, Robinson R, Jakubowski E (2007). Strategic purchasing to improve health system performance: Key issues and international trends. Healthc Pap.

[6] Kibria A, Mancher M, McCoy MA, Graham RP, Garber AM, Newhouse JP (2013). Variation in health care spending: Target decision making, not geography.

[7] McIntyre D (2007). Learning from experience: Health care financing in low-and middle-income countries.

[8] Mathauer I, Dale E, Meessen B, Organization WH (2017). Strategic purchasing for universal health coverage: Key policy issues and questions: A summary from expert and practitioners’ discussions.

[9] Gosden T, Forland F, Kristiansen I (2000). Capitation, salary, fee-for-service and mixed systems of payment: Effects on the behaviour of primary care physicians. Cochrane Libr.

[10] Nicholson D, Yates R, Warburton W, Fontana G (2015). Delivering universal health coverage: A guide for policymakers. Report of the WISH Universal Health Coverage Forum.

[11] Langenbrunner J, Cashin C, O’Dougherty S (2009). Designing and implementing health care provider payment systems: How-to manuals.

[12] Cashin C (2015). Assessing health provider payment systems: A practical guide for countries working toward universal health coverage.

[13] Rosenthal MB (2006). Pay for performance: A decision guide for purchasers. Agency Healthcare Res Qual (AHRQ).

[14] Rhys G, Beerstecher HJ, Morgan CL (2010). Primary care capitation payments in the UK. An Observational Study. BMC Health Serv Res.

[15] Barnum H, Kutzin J, Saxenian H (1995). Incentives and provider payment methods. Int J Health Plann Manag.

[16] Agyei-Baffour P, Oppong R, Boateng D (2013). Knowledge, perceptions and expectations of capitation payment system in a health insurance setting: A repeated survey of clients and health providers in Kumasi, Ghana. BMC Public Health.

[17] Munge K, Mulupi S, Chuma J (2015). A critical analysis of the purchasing arrangements in Kenya: The case of the National Hospital Insurance Fund, Private and Community-based health insurance: RESYST.

[18] Marshall L, Charlesworth A, Hurst J (2014). The NHS payment system: Evolving policy and emerging evidence.

[19] Kroneman M (2011). Paying general practitioners in Europe.

[20] Lagarde M, Powell-Jackson T, Blaauw D (2010). Managing incentives for health providers and patients in the move towards universal coverage.

[21] Lagarde M, Blaauw D (2017). Physicians’ responses to financial and social incentives: A medically framed real effort experiment. Soc Sci Med.

[22] Krasnik A, Groenewegen PP, Pedersen PA (1990). Changing remuneration systems: Effects on activity in general practice. BMJ.

[23] Mohammed S, Souares A, Bermejo JL, Sauerborn R, Dong H (2014). Performance evaluation of a health insurance in Nigeria using optimal resource use: Health care providers perspectives. BMC Health Serv Res.

[24] Hsu P-F (2014). Does a global budget superimposed on fee-for-service payments mitigate hospitals’ medical claims in Taiwan?. Int J Health Care Finance Econ.

[25] Reschovsky JD, Hadley J, Landon BE (2006). Effects of compensation methods and physician group structure on physicians’ perceived incentives to alter services to patients. Health Serv Res.

[26] Alqasim KM, Ali EN, Evers SM, Hiligsmann M (2016). Physicians’ views on pay-for-performance as a reimbursement model: A quantitative study among Dutch surgical physicians. J Med Econ.

[27] Basinga P, Gertler PJ, Binagwaho A, Soucat AL, Sturdy J, Vermeersch CM (2011). Effect on maternal and child health services in Rwanda of payment to primary health-care providers for performance: An impact evaluation. Lancet.

[28] Chen T-T, Lai M-S, Chung K-P (2015). Participating physician preferences regarding a pay-for-performance incentive design: A discrete choice experiment. Int J Qual Health Care.

[29] Federman AD, Woodward M, Keyhani S (2010). Physicians’ opinions about reforming reimbursement: Results of a national survey. Arch Intern Med.

[30] Feng Z, Grabowski DC, Intrator O, Zinn J, Mor V (2008). Medicaid payment rates, case-mix reimbursement, and nursing home staffing—1996-2004. Med Care.

[31] Harrington C, Swan JH, Carrillo H (2007). Nurse staffing levels and Medicaid reimbursement rates in nursing facilities. Health Serv Res.

[32] Olafsdottir AE, Mayumana I, Mashasi I (2014). Pay for performance: An analysis of the context of implementation in a pilot project in Tanzania. BMC Health Serv Res.

[33] Robyn PJ, Bärnighausen T, Souares A (2012). Health worker preferences for community-based health insurance payment mechanisms: A discrete choice experiment. BMC Health Serv Res.

[34] James Tufano M, Conrad DA, Sales A (2001). Effects of compensation method on physician behaviors. Am J Manag Care.

[35] Wang J, Hong SH, Meng S, Brown LM (2011). Pharmacists’ acceptable levels of compensation for MTM services: A conjoint analysis. Res Soc Adm Pharm.

[36] Agyepong IA, Aryeetey GC, Nonvignon J (2014). Advancing the application of systems thinking in health: Provider payment and service supply behaviour and incentives in the Ghana National Health Insurance Scheme–a systems approach. Health Res Policy Syst.

[37] Koduah A, van Dijk H, Agyepong IA (2016). Technical analysis, contestation and politics in policy agenda setting and implementation: The rise and fall of primary care maternal services from Ghana’s capitation policy. BMC Health Serv Res.

[38] Sieverding M, Onyango C, Suchman L (2018). Private healthcare provider experiences with social health insurance schemes: Findings from a qualitative study in Ghana and Kenya. PLoS One.

[39] (2018). Critical Appraisal skills programme (CASP) Check lists.

[40] Hannes K (2011). Critical appraisal of qualitative research.

[41] Clemens J, Gottlieb JD (2017). In the shadow of a giant: Medicare’s influence on private physician payments. J Polit Econ.

[42] Ellis RP, Miller MM (2007). Provider payment methods and incentives. Health Syst Policy, Finance, Organ.

[43] Perry BJ (2017). Essays on the impact of supply-side regulation in US health care markets.

[44] Organization WH (2014). Paying for performance in health care implications for health system performance and accountability: Implications for health system performance and accountability.

[45] Mannion R, Davies HT (2008). Payment for performance in health care. BMJ.

[46] Agyepong IA, Nagai RA (2011). “We charge them; otherwise we cannot run the hospital” front line workers, clients and health financing policy implementation gaps in Ghana. Health Policy.

[47] Yett DE, Der W, Ernst RL, Hay JW (1983). Physician pricing and health insurance reimbursement. Health Care Financ Rev.

[48] Boyanagari M, Boyanagari VK (2018). Perceptions and experiences of healthcare providers and beneficiaries on the national health insurance scheme of Rashtriya Swasthya BimaYojana (RSBY) in a taluk of south Indian state of Karnataka. Clin Epidemiol Global Health.

[49] Dalinjong PA, Laar AS (2012). The national health insurance scheme: Perceptions and experiences of health care providers and clients in two districts of Ghana. Heal Econ Rev.

[50] Yuan B, He L, Meng Q, Jia L (2017). Payment methods for outpatient care facilities. Cochrane Libr.

[51] Khullar D, Chokshi DA, Kocher R (2015). Behavioral economics and physician compensation—promise and challenges. N Engl J Med.

[52] Kaur M, Hall S, Attawell K (2001). Medical supplies and equipment for primary health care: A practical resource for procurement and management.

[53] Toward CW Assessing health provider payment systems.

[54] Hussey PS, Mulcahy AW, Schnyer C, Schneider EC (2012). Closing the quality gap: Revisiting the state of the science (vol. 1: bundled payment: effects on health care spending and quality).

[55] Botje D, ten Asbroek G, Plochg T (2016). Are performance indicators used for hospital quality management: A qualitative interview study amongst health professionals and quality managers in the Netherlands. BMC Health Serv Res.

[56] Kruse GB, Polsky D, Stuart EA, Werner RM (2012). The impact of hospital pay-for-performance on hospital and Medicare costs. Health Serv Res.

[57] Shen GC, Nguyen HTH, Das A (2017). Incentives to change: Effects of performance-based financing on health workers in Zambia. Hum Resour Health.

[58] Thomas J, Harden A (2008). Methods for the thematic synthesis of qualitative research in systematic reviews. BMC Med Res Methodol.

